# RNF6 promotes chronic myelogenous leukemia cell proliferation and migration by stabilizing vimentin via multiple atypical ubiquitinations

**DOI:** 10.1016/j.gendis.2023.04.004

**Published:** 2023-05-10

**Authors:** Hongxia Zhang, Yueya Zhong, Yuanming He, Yujia Xu, Ying Ren, Haixia Zhuang, Tong Sun, Zhigang Zhu, Xinliang Mao

**Affiliations:** a Institute of Clinical Pharmacology, Science and Technology Innovation Center, Guangzhou University of Chinese Medicine, Guangzhou, Guangdong 510403, China; b Guangdong Provincial Key Laboratory of Protein Modification and Degradation, School of Basic Medical Sciences, Guangzhou Medical University, Guangzhou, Guangdong 511436, China; c Department of Pharmacology, College of Pharmaceutical Sciences, Soochow University, Suzhou, Jiangsu 215123, China; d Division of Hematology & Oncology, Department of Geriatrics, Guangzhou First People's Hospital, College of Medicine, South China University of Technology, Guangzhou, Guangdong 510180, China

Chronic myelogenous leukemia (CML) is a malignancy from bone marrow myeloid stem cells mainly driven by the fusion gene BCR-ABL. In addition to BCR-ABL, other genes including RNF6 are also dysregulated in CML cells.^1^ RNF6, a ubiquitin ligase of the RING family, promotes various cancer cell proliferation, chemoresistance, and tumor growth *in vivo* by targeting various proteins for ubiquitination and degradation, including SHP1, TLE3, FOXA1, and MAD1.^2^ However, its specific mechanism in CML is not known.

To find out the specific protein substrates of RNF6 in CML cells, we performed an affinity-purification coupled LC/MS/MS assay and found that vimentin (VIM), a major constituent of the intermediate filament family of proteins and a key regulator of tumor growth, invasion, and poor prognosis,^3^ is an interacting partner of RNF6 which was further confirmed in CML and HEK293T cells (Fig. S1A, B; Fig. 1A, B). Given RNF6 is a ubiquitin ligase to regulate the stability of specific proteins, we next examined VIM protein in the presence or absence of RNF6. The results showed that overexpression of RNF6 increased VIM protein, in contrast, knockdown or knockout of RNF6 by its specific shRNA or sgRNA brought down VIM protein in both K562 and KMB5, two typical CML cell lines (Fig. 1C, D; Fig. S1A, B). Notably, RNF6 showed no activity on HDAC2 stability (Fig. 1C, D), another potential substrate found in the MS assay (Fig. S1A, C). Moreover, RNF6 did not alter the mRNA level of VIM (Fig. S2C, D). To further evaluate the effect of RNF6 on VIM protein stability, HEK293T cells were transfected with VIM alone or along with RNF6 plasmids, followed by the treatment of cycloheximide, an inhibitor of protein synthesis *de novo*. The subsequent IB analysis revealed that in the presence of RNF6, the half-life of VIM was markedly increased (Fig. 1E; Fig. S2E). Moreover, the correlation analysis of VIM and RNF6 proteins in various cell lines also found that the VIM protein level was highly associated with RNF6 (Fig. 1F; Fig. S2F). All the above studies collectively concluded that RNF6 up-regulated VIM at the post-translational, not at the transcriptional level.

**Figure 1:**
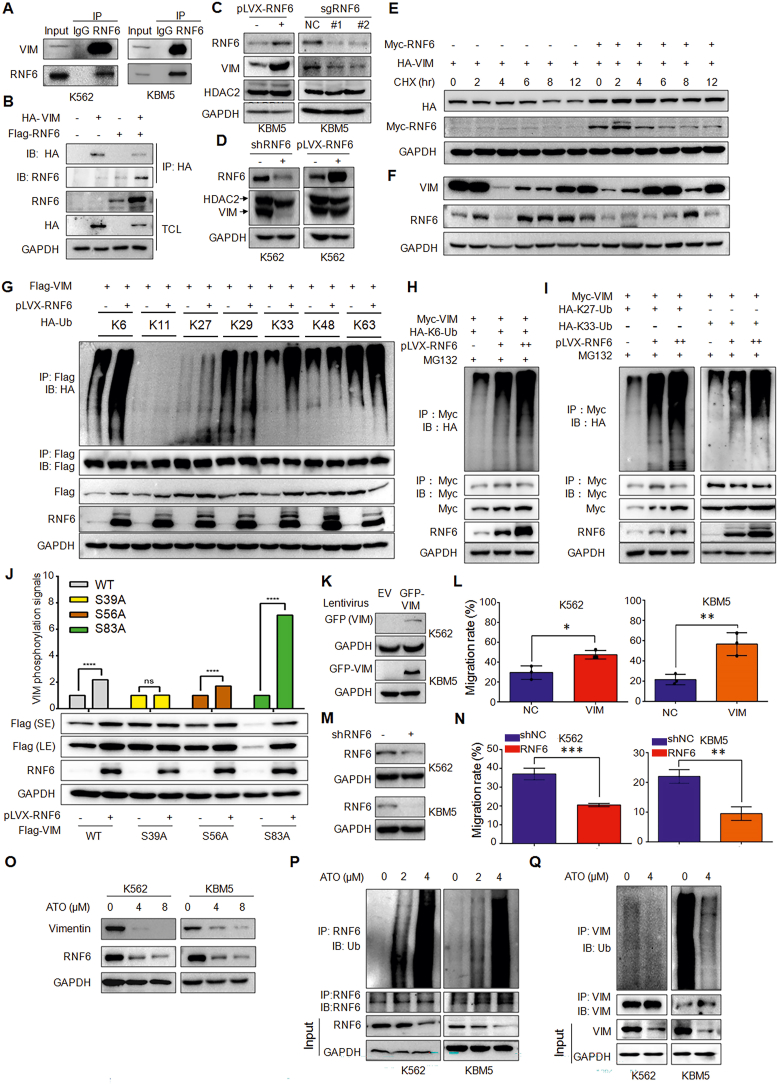
RNF6 mediates VIM for multiple atypical polyubiquitinations and stabilizes VIM to promote CML cell migration. **(A)** CML cell lysates were subject to IP/IB assays as indicated. **(B)** VIM and RNF6 plasmids were co-transfected into HEK293T cells, followed by IP/IB assays. **(C)** KBM5 cells were infected with pLVX-RNF6 or sgRNF6 lentivirus. Ninety-six hours later, cell lysates were prepared for IB with specific antibodies. **(D)** K562 cells were infected with pLVX-RNF6 or RNF6 shRNAs lentivirus. Ninety-six hours later, cell lysates were prepared for IB assay against specific proteins as indicated. **(E)** HEK293T cells were transfected with HA-VIM, with or without Myc-RNF6 for 24 h followed by CHX treatment for indicated duration. Cell lysates were then prepared for IB assay. **(F)** The whole-cell lysates from various cell lines were subject to IB assays against RNF6 and VIM. **(G)** HEK293T cells were co-transfected with Flag-VIM, different linkages of HA-Ubiquitin (wild-type, K6-, K11-, K27-, K29-, K33-, K48-, K63-Ub), with or without pLVX-RNF6 for 48 h. The cell lysates were subject to IP/IB assays as indicated. **(H, I)** HEK293T cells were transfected with Myc-VIM, K6-Ub **(H)** or K27Ub or K33-Ub **(I)** and increased pLVX-RNF6 for 48 h. The cell lysates were subjected to IP/IB as indicated. **(J)** The 293T cells were co-transfected with Flag-Vimentin mutant (wild-type, S39A, S56A, S83A) plasmids in the absence or presence of pLVX-RNF6. Cell lysates were subjected to IB with the indicated antibodies. **(K)** K562 and KBM5 cells were infected with lentiviral vimentin for 96 h followed by IB assays. **(L)** Cells infected with lentiviral VIM as shown in (K) were subjected to Transwell migration assays. **(M)** K562 and KBM5 cells were infected with lentiviral shRNF6 for 96 h followed by IB assays. **(N)** Cells infected with lentiviral shRNF6 as shown in (M) were subjected to Transwell migration assays. **(O)** K562 and KBM5 cells were treated with ATO for 24 h and cell lysates were then subjected to IB assays against RNF6 and VIM. **(P, Q)** K562 and KBM5 cells were treated with ATO for 12 h and cell lysates were then subjected to immunoprecipitation with an anti-VIM antibody and IB for K48-linked Ub (P) and K63-linked ubiquitination (Q).

Given RNF6 is a ubiquitin ligase and it stabilizes VIM protein, we wondered whether the underlying mechanism was associated with VIM ubiquitination. To this end, RNF6 and Ub plasmids were transfected into HEK293T cells followed by IP and IB assays to evaluate the VIM ubiquitination level. The results showed that RNF6 promoted VIM polyubiquitination (Fig. S3A). Moreover, when RNF6 was knocked down, VIM ubiquitination was significantly reduced (Fig. S3B). Because RNF6 is a RING family ubiquitin ligase and its activity is dependent on the RING domain,^4^ we next further discussed whether the RING domain was essential for RNF6 to ubiquitinate VIM. To this end, RNF6 or its RING-lacking mutant (ΔRING) was co-transfected with VIM, the following IP/IB assay showed that RNF6 again increased VIM ubiquitination, but the ΔRING mutant failed to increase VIM ubiquitination (Fig. S3C). This result thus suggested that RNF6 mediated VIM ubiquitination in a manner dependent on its RING domain.

Given the diversity of ubiquitination forms and different results from specific ubiquitination forms that have been found in RNF6,^2^ we next wondered which specific form(s) of ubiquitination on VIM mediated by RNF6. To this end, VIM plasmids were co-transfected with wild-type (wt) and single lysine-containing ubiquitin plasmids with or without RNF6, the subsequent IP/IB assays revealed that the K6-, K27- and K33-ubiquitination on VIM was increased in the presence of RNF6 (Fig. 1G). Notably, K11- and K48-linked polyubiquitination, the major ubiquitination form for protein degradation, was not seen in VIM by RNF6 (Fig. 1G). Moreover, RNF6 increased the K6-, K27-, and K-33-linked polyubiquitinations on VIM in a concentration-dependent manner (Fig. 1H, I). Therefore, all these results demonstrated that RNF6 promotes VIM for multiple forms of polyubiquitination.

VIM is activated by phosphorylation, especially at Ser39, to increase cancer cell motility and invasion.^5^ To find out the effects of RNF6 on VIM activity, we knocked down or overexpressed RNF6 in CML cells, and the subsequent IB analysis revealed that RNF6 up-regulated VIM, along with its Ser39 phosphorylation (Fig. S4A, B). Furthermore, when VIM was mutated at several common phosphorylation sites, including S39A, S56A, and S83A, it turned out that RNF6 failed to stabilize S39A VIM but increased the levels of the WT and the S56A and S83A VIM (Fig. 1J). Interestingly, RNF6 binding to S39A VIM was reduced (Fig. S4C), further suggesting that RNF6 acts on phosphorylated VIM. This finding, therefore, suggests RNF6 might specifically prevent the degradation of VIM with S39 phosphorylation; it also implicates RNF6 promotes VIM for its oncogenic activity.

Given that RNF6 promotes VIM stability and increases its S39 phosphorylation, RNF6 and VIM might contribute to CML cell proliferation and migration. To this end, we infected CML cell lines with VIM lentivirus (Fig. 1K) and indeed found that overexpression of VIM increased CML cell migration (Fig. 1L). Moreover, VIM also promoted CML cell proliferation (Fig. S5A). Consistent with these observations, RNF6 knockdown (Fig. 1M) reduced CML cell migration (Fig. 1N) and proliferation (Fig. S5B). Moreover, the effects of shRNF6 on CML cell proliferation and migration were partly prevented by lentiviral VIM (Fig. S5C, D). These findings suggest that the action of RNF6 on CML cells is partly via VIM.

We recently found that RNF6 could be degraded via autoubiquitination by the anti-CML agent nilotinib.^4^ In the drug screen, we found that arsenic trioxide (ATO) was able to down-regulate both RNF6 and VIM (Fig. 1O) and suppressed CML cell proliferation (Fig. S6A), and the overexpression of either RNF6 or VIM could rescue the cleavage and activation of PARP and caspase-3 induced by ATO (Fig. S6B, C), the hallmarks of apoptosis, suggesting ATO induces CML cell death partly via the RNF6/VIM axis. Furthermore, we found ATO promoted RNF6 polyubiquitination but prevented VIM for polyubiquitination (Fig. 1P, Q). We further found that ATO showed no activity on K6-, K48- and K63-linked ubiquitination of VIM (Fig. S6D, E), but reduced the K27- and K33-linked ubiquitination (Fig. S6E). These findings thus suggest that ATO induced MM cell apoptosis by targeting the RNF6/VIM axis.

Our previous report found that RNF6 is a downstream gene of the transcription factor PBX1 in CML cells.^1^ The PBX1/RNF6 axis contributes to CML proliferation, chemoresistance, and tumor growth *in vivo.*^1^ The present study further revealed a molecular mechanism of RNF6 in promoting CML cell migration and proliferation. We demonstrated that RNF6 binds to and mediates VIM for multiple atypical polyubiquitinations including K6-, K27-, and K33-linked forms, and stabilizes and activates VIM in CML cells. Although previous reports have documented RNF6 promotes the development of solid cancers by activating various oncogenic signaling pathways, including SHP1/STAT3, Wnt/β-catenin, or androgen receptors via K63-, K48-, and K27-linked polyubiquitination.^2^ The present study adds novel ubiquitination forms mediated by RNF6 and it will help to understand the pathophysiology of RNF6 and VIM in CML.

In conclusion, our study identifies RNF6 as a novel ubiquitin ligase of VIM in CML cells. RNF6 mediates multiple atypical ubiquitinations on VIM and these modifications prevent VIM from degradation. The RNF6/VIM axis promotes CML cell proliferation and migration. Targeting the RNF6/VIM axis could be a novel strategy for CML treatment.

## Author contributions

Xinliang Mao: conceptualization, methodology, funding, and manuscript writing; Zhigang Zhu: data analysis and funding; Hongxia Zhang: investigation, manuscript drafting; Yueya Zhong, Yuanming He, Ying Ren, Yujia Xu, Haixia Zhuang, and Tong Sun: investigation.

## Conflict of interests

The authors declare that they have no conflict of interests.

## Funding

This project was partly supported by The 10.13039/501100001809National Natural Science Foundation of China (No. 81770154, 81970194, and 82170176), the 10.13039/501100012166National Key Research and Development Program of China (No. 2022YFC2705003), 10.13039/100009659Guangzhou Medical University Discipline Construction Funds (Basic Medicine) (China) (No. JCXKJS2022A05), and Guangzhou Key Discipline of Medicine (Geriatric Medicine) (China) (No. ZDXK202103).
