# Supplementary material for: RNF6 promotes chronic myelogenous leukemia cell proliferation and migration by stabilizing vimentin via multiple atypical ubiquitinations
Source: Genes Dis. 2023 May 10;11(1):87–90. doi: 10.1016/j.gendis.2023.04.004 (PMC10425833; doi:10.1016/j.gendis.2023.04.004)
Supplement: Multimedia component 1 [file mmc1.pdf]

Supplemental Materials for the paper

"RNF6 promotes chronic myelogenous leukemia cell proliferation and migration by stabilizing vimentin via multiple atypical ubiquitinations"

Material and methods

Cells and cell culture

Chronic myelogenous leukemia cell lines (K562, KBM5), acute leukemia cell lines (OCI-AML2, HEL, HL-60, NB4, THP-1, and Jurkat), multiple myeloma cell lines (KMS11, LP1, OPM2, and RPMI-8226) and Human embryonic kidney cells lines (HEK293T) were purchased from American Type Culture Collection (ATCC, Manassas, Virginia, USA) or maintained in the lab. Cells were cultured in Iscove's modified Dulbecco medium (IMDM, Hyclone), RPMI 1640 medium (Hyclone), or Dulbecco's high glucose modified Eagle's medium (DMEM, Hyclone), respectively. All cells were cultured at 37℃ with 5% CO2 in an appropriate medium supplemented with 10% fetal bovine serum (ExCell Bio, Inc., Shanghai, China), 1% Penicillin-streptomycin (GIBCO).

Plasmids

The plasmids for RNF6 and truncates were generated as described previously [8]. The lentivirus of RNF6 and USP7 were prepared as reported previously [16]. The VIM coding sequence was retrieved from the NCBI website (http://www.ncbi.nlm.nih.gov) and the full-length human VIM cDNA was amplified from K562 cells by PCR. The

VIM coding sequence were subcloned into a pcDNA3.1 vector carrying a FLAG or a Myc or a HA tag, resulting in Flag-VIM, Myc -VIM, HA-VIM, respectively. The lentiviral VIM fused with HA-GFP was generated in the pLVX frame plasmid. The lentiviral sgRNAs against VIM (sgVIM) and the negative control were purchased from Miaolingbio. Inc (Wuhan, China). The target sequences of sgVIM#1, sgVIM#2 and sgVIM #3 were 5'-tcctaccgcaggatgttcgg -3', 5′-gaactcggtgttgatggcgt -3' and 5'-caacgacaaagcccgcgtcg-3', respectively. The HA-Ub and HA-Ub mutants were generated in the lab [16]. The preparation of lentiviral plasmids was described previously [16].

Antibodies and chemicals

Antibodies against USP7, K48-Ub, and K63-Ub were purchased from Cell Signaling Technologies, Inc. (Danvers, MA, USA). The antibodies against RNF6, VIM, and GAPDH were obtained from Proteintech (Wuhan, China). The monoclonal antibodies including anti-Flag, anti-HA, and anti-Myc were obtained from Medical and Biological Laboratories Co., Ltd (Nagoya, Japan). The anti-Ub antibody was purchased from Santa Cruz Biotechnology, Inc. (Santa Cruz, CA, USA). HRP-labeled goat anti-mouse and goat anti-rabbit IgG (H + L) antibodies were purchased from Beyotime Institute of Biotechnology (Nantong, China). MG132 and P5091 were obtained from Santa Cruz Biotechnology and Selleck Chemicals Inc (Houston, TX), respectively. Cycloheximide (CHX) was provided by Sigma-Aldrich Chemicals (St.

Louis, MO, USA). Arsenic trioxide (ATO) was purchased from TMRM Chemicals Co. Ltd (Jiangsu, China).

MTT assay

Cell viability was evaluated by the MTT (3-(4, 5-dimethylthiazol-2-yl)- 2,5-diphenyl tetrazolium bromide) assay as described previously [17].

Reverse-transcription polymerase chain reaction (RT-PCR)

Total RNA from cultured cells was extracted using RNAeasy™ Animal RNA Isolation Kit (Beyotime Biotech) following the manufacturer's instructions. Reverse transcription was carried out with 1.5μg of purified RNA using MonScript™ RTIII Super Mix with dsDNase (Two-Step) (TransGen Biotech Co., Ltd (Beijing, China) according to the protocol provided by the manufacturer. The synthesized cDNA was amplified by PCR using the following primers: for RNF6, Forward 5'-CATCAGTGGCTCTTCGGTCA-3' and Reverse 5'-GACCCGGAGCTTTTCACCTTTAGTT-3'; for VIM, Forward 5'-GCCAGGCAAAGCAGGAGT-3' and Reverse 5'-GGGTATCAACCAGAGGGAGT-3'; for GAPDH, Forward 5'-AATCCCATCACCATCTTCC-3' and Reverse 5'-ATGCTCATAGTGCCTGGTGG-3'. Reaction cycling conditions were 5 min at 94 °C, followed by 30 cycles at 94 °C for 30 s, 55 °C for 30 s,72 °C for 40 s, and 1

cycle at 72 °C for 5 min. The PCR products were visualized by GoldView II Nuclear Staining Dyes following electrophoresis on 1% agarose gels.

Gene transfection

HEK293T cells at the log phase with 60%-80% confluence were subjected to gene transfection using polyethyleneimine (PEI, Sigma-Aldrich Co., St. Louis, MO, USA) as a gene delivery carrier as described previously [18]. siRNAs against RNF6 and a control scrambled siRNA were synthesized by GenePharma (Shanghai, China). The detailed specific sequences targeting RNF6 (siRNF6) were described previously[16]. Small interfering RNA (siRNA) was transfected into HEK293T cells by Lipofectamine 2000 (Invitrogen) according to the manufacturer's instructions.

Immunoblotting (IB)

Total proteins were extracted from treated cells using a 0.5% sodium dodecyl sulfate (SDS)-containing protein lysis buffer. Protein concentrations in the extracts were measured with a Bicinchoninic acid assay (Beyotime, Nantong, China) and were made equal in different samples with an extraction reagent. Immunoblotting analyses were performed as previously described [16].

Immunoprecipitation (IP)

The detailed protocol was described previously [16]. Specifically, cells of interest were collected and lysed in a lysis buffer containing 50 mM HEPES, 150 mM NaCl,1.5

mM $MgCl_2$, 10 mM NaF,1 mM EGTA, 10% Glycerol, 1% TritonX-100 and a protease inhibitor cocktail (Cell Signaling Technology). After centrifugation at 12,000 rpm for 20 min at 4 °C, the supernatants (1 mg) were pre-incubated with Protein A+G agarose beads (Beyotime Biotech Co. Ltd) for 1 hr at 4 °C. After several wash, the supernatants were subjected to incubation overnight at 4 °C with specific antibodies as needed. The proteins were then incubated with Protein A+G 2hr at room to precipitate proteins of interest. After centrifugation at low speed at cold, the beads were collected to elute proteins in 2× SDS sample loading. Eluted proteins and whole-cell lysates were loaded and subjected to SDS–polyacrylamide gel electrophoresis (SDS–PAGE) and IB assays against specific antibodies.

Affinity purification-coupled tandem mass spectrometry (AP/MS/MS) assay

To find proteins that interacted with RNF6, HEK293T cells were transfected with a Flag–RNF6ΔRING plasmid or empty vector for 36h before being treated with MG132 (20 μM) for another 4 h. The cell lysates were subjected to IP against an anti-Flag antibody. After gentle washing, the immunoprecipitated proteins were separated by SDS-PAGE, followed by silver staining (Beyotime Biotech). The protein bands from the gel were excised and processed for high performance liquid chromatography and tandem mass spectrometry (MS/MS) analysis as described previously [16]. The data processing protocol was described previously [16].

Cycloheximide Chase Assay

To evaluate whether RNF6 regulates the stability of VIM protein, HEK293T cells were transfected with Myc-RNF6 and HA-VIM. 24 h later, cells were treated with cycloheximide (100 μg/ml, Sigma-Aldrich) for a specific period before preparing for SDS-PAGE and IB analyses.

Cell migration assay

Cell migration assay was performed using 24-well culture plates containing transparent PET membrane inserts (Corning, NY, USA) as described in the manufacturer's protocol [19]. Puromycin-resistant cells (10×10^4) were seeded into the top chamber, which contained 100 μL serum-free medium, and the bottom chamber contained 600 μL medium with 10% FBS. After 48 h of incubation, the numbers of cells that migrated through the membrane were quantified for statistical analysis. Each experiment was performed in triplicate.

Statistical analysis

Two-tailed Student's t-test and two-way ANOVA test were used to compare two groups in the studies. All statistical tests were two-sided, and a *p*-value <0.05 was considered statistically significant. Statistical significance between groups was analyzed with GraphPad Prism 5.0.

Supplemental Figures

A RNF6 binding partner candidates

Accession	Protein Name	∑Coverage	∑#Proteins	Unique peptide	Coverage A2	Coverage B2	#AAs	MV[kDa]
Q92769	HDAC2	6.77	3	1	6.77	0	458	52
P08670	Vimentin	22.1	5	7	22.1	0	466	53.6

B P08670 · VIME_HUMAN, 7 Unique peptides, $p<0.05$

MSTRSVSSSS YRRMFGGPGT ASRPSSSRSY VTTSTRTYSL GSALRPSTSR **SLYASSPGGV**
YATRSSAVRL RSSVPGVRLL QDSVDFSLAD AINTEFKNTR **TNEKVELQEL** **NDR**FANYIDK
VRFLEQQNKI LLAELEQLKG QGKSRLGDLY EEEMRELRRQ VDQLTNDKAR VEVERDNLAE
DIMRLREK**LQ** **EEMLQREEAE** **NTLQSFR**QDV DNASLARLDL ERKVESLQEE IAFLKKLHEE
EIQELQAQIQ EQHVQIDVDV SKPDLTAALR DVRQQYESVA AKNLQEAEEW YKSK**FADLSE**
AANRNNDALR QAKQESTEYR R**QVQSLTCEV** **DALK**GTNESL **ERQMREMEEN** **FAVEAANYQD**
TIGRLQDEIQ NMKEEMARHL REYQDLLNVK MALDIEIATY RKLLEGEESR ISLPLPNFSS
LNLR**ETNLDS** **LPLVDTHSK**R TLLIKTVETR DGQVINETSQ HHDDLE

C > Q92769 · HDAC2_HUMAN, 1 Unique peptide, $p < 0.05$

MAYSQGGGKK KVCYYYDGDI GNYYYGQGHP MKPHRIRMTH NLLLNYGLYR KMEIYRPHKA
TAEEMTKYHS DEYIKFLRSI RPDNMSEYSK QMQRFNVGED CPVFDGLFEF CQLSTGGSVA
GAVKLNRQQT DMAVNWAGGL HHAKKSEASG FCYVNDIVLA ILELLKYHQR VLYIDIDIHH
GDGVEEAFYT TDRVMTVSFH KYGEYFPGTG DLRDIGAGKG KYYAVNFPMR DGIDDESYGQ
IFKPIISKVM EMYQPSAVVL QCGADSLSGD RLGCFNLTVK GHAKCVEVVK TFNLPLLMLG
GGGYTIRNVA RCWTYETAVA LDCEIPNELP YNDYFEYFGP DFK**LHISPSN** **MTNQNTPEYM**
EKIKQRLFEN LRMLPHAPGV QMQAIPEDAV HEDSGDEDGE DPDKRISIRA SDKRIACDEE
FSDSEDEGEG GRRNVADHKK GAKKARIEED KKETEDKKTD VKEEDKSKDN SGEKTDTKGT
KSEQLSNP

Supplemental Fig. 1. VIM was identified by mass spectrometry in the RNF6-interaction proteome. A, the summary of potential RNF6 binding proteins VIM and HDAC2. B, Unique peptides in VIM identified by MS in the RNF6 co-immunoprecipitated complex were highlighted in red. C, Unique peptides in HDAC2 identified by MS in the RNF6 co-immunoprecipitated complex were highlighted in red.

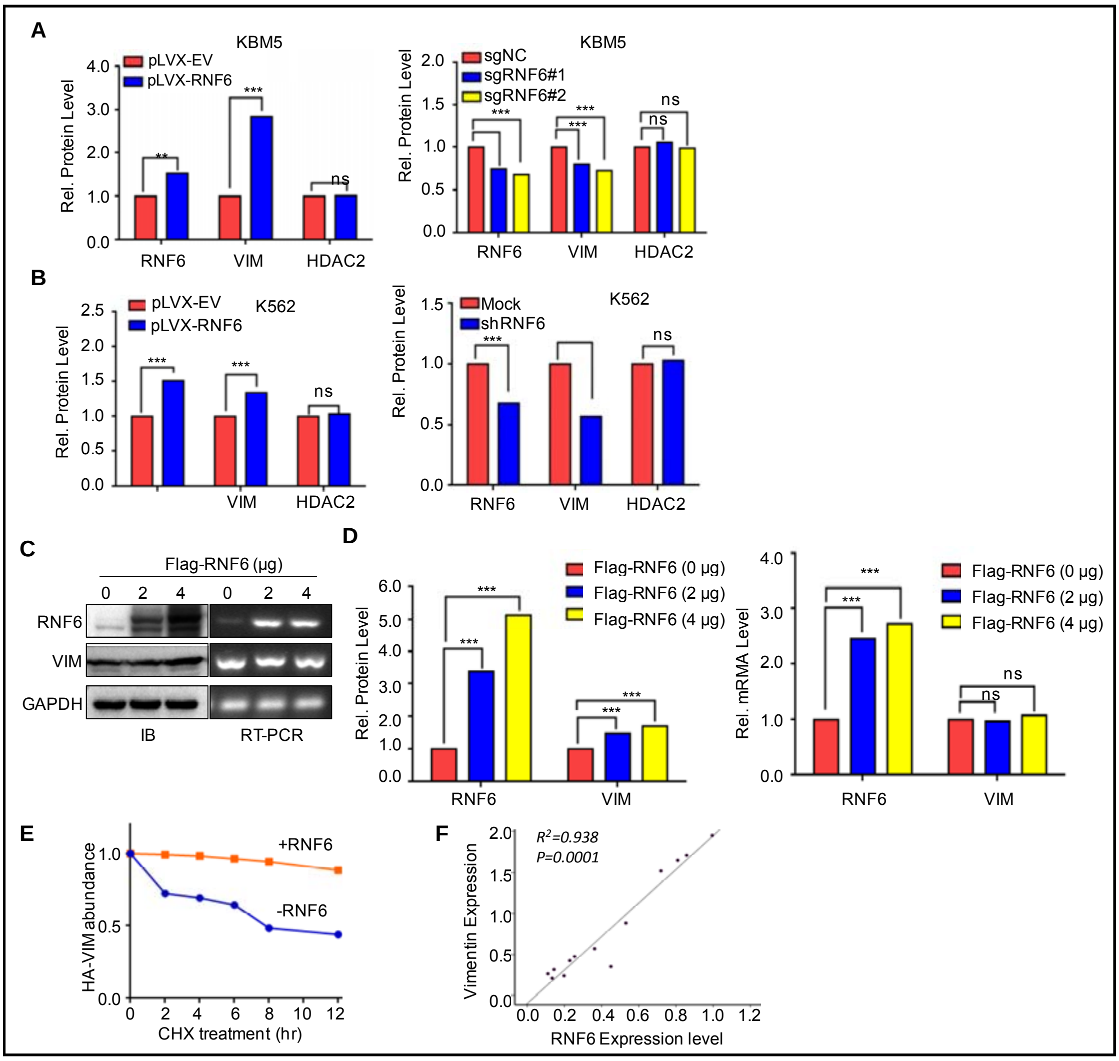

Supplemental Fig. 2. RNF6 stabilizes vimentin at the protein level but not the mRNA level. A, KBM5 cells were infected with pLVX-RNF6 or sgRNF6 lentivirus. Ninety-six hours later, cell lysates were prepared for IB with specific antibody. The relative expression levels of VIM and RNF6 were represented based on the densitometric analysis of blot bands as shown in Fig. 1C. B, K562 cells were infected with pLVX-RNF6 or RNF6 shRNAs lentivirus. Ninety-six hours later, cell lysates were prepared for IB assay against specific proteins. The relative expression levels of VIM and RNF6 were represented based on the densitometric analysis of blot bands as shown in Fig. 1D. C, HEK293T cells were transfected with Flag-RNF6 plasmids for

48 h, followed by IB and RT-PCR assays as indicated. D, The relative expression levels of VIM and RNF6 were represented based on the densitometric analysis of bands as shown in C. E, HEK293T cells were transfected with HA-VIM, with or without Myc-RNF6 for 24 h followed by CHX treatment for indicated duration. Cell lysates were then prepared for IB assays.VIM expression levels were analyzed against GAPDH based on the IB result in Fig. 1E. F, the whole-cell lysates from normal HEK293T, various leukemia and multiple myeloma cell lines were subject to IB assays against RNF6 and VIM. The correlation between RNF6 and VIM was analyzed based on the IB blots as shown in Fig. 1F.

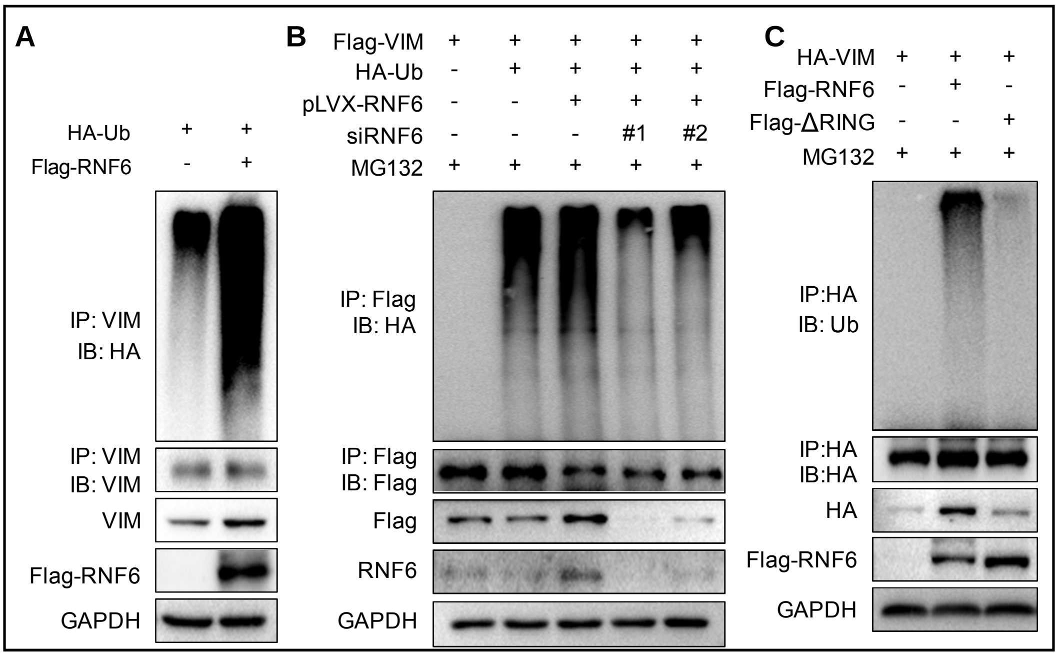

Supplemental Fig. 3. RNF6 induces VIM ubiquitination requiring its RING domain. A, HEK293T cells were co-transfected with HA-Ubiquitin and Flag-RNF6. The cell lysates were subjected to IP/IB with specific antibodies as indicated. B, HEK293T cells were co-transfected Flag-VIM, HA-Ub, and pLVX-RNF6 with or without siRNF6, followed by IP/IB assays as indicated. C, HEK293T cells were co-transfected with HA-VIM and Flag-ΔRING or Flag-RNF6 plasmids for 48 h, followed by MG132 treatment for 4 h before being subjected to IP/IB assays.

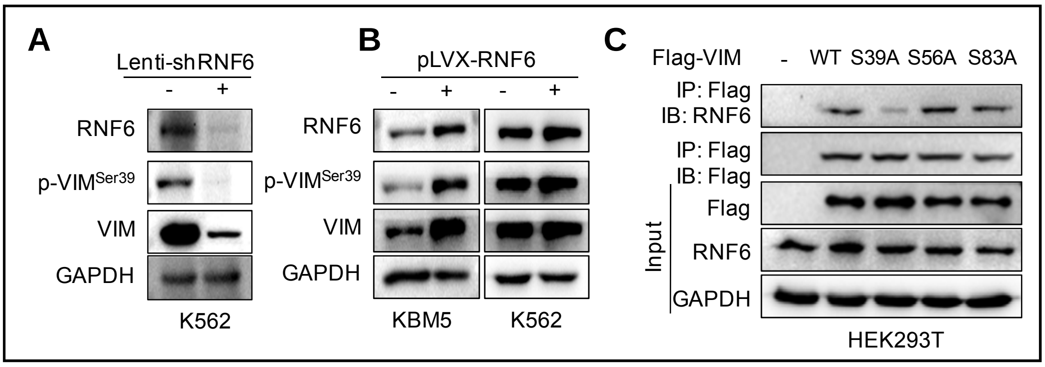

Supplemental Fig. 4. RNF6 increases and binds to phospho-VIM at S39. A, K562 cells were infected with lentiviral RNF6 shRNF6 for 96 h followed by IB assays. B, Lentiviral RNF6 was infected into K562 and KBM5 cell lines for 96 h. The cell lysates were then prepared for immunoblotting assay against specific antibodies as indicated. C, HEK293T cells were transfected with Flag-tagged VIM and its mutants for 24 h, cell lysates were then prepared for IP/IB assays as indicated.

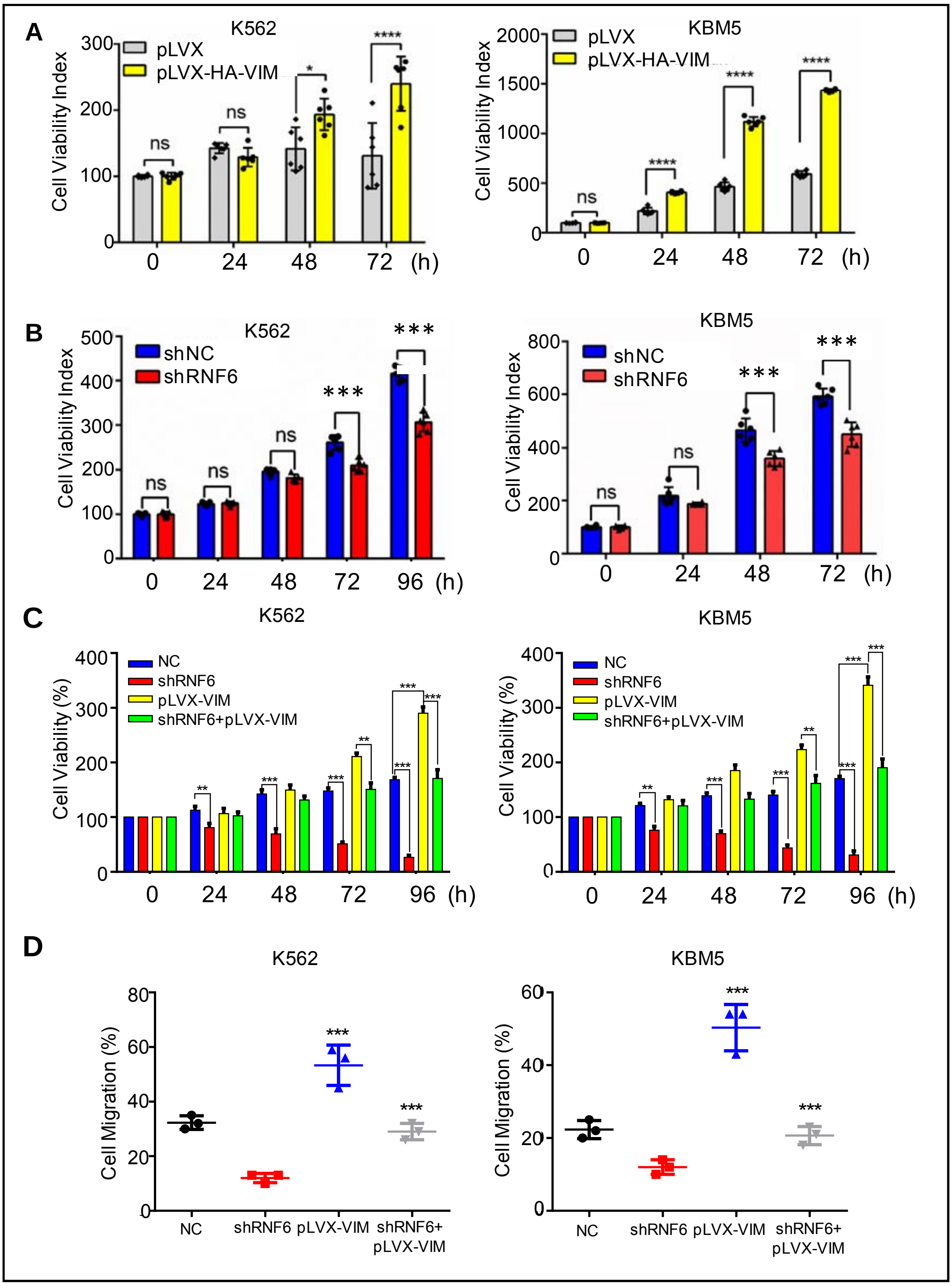

Supplemental Fig. 5. RNF6 and Vimetin promote CML cell proliferation. A, CML cells were infected with lentiviral vimentin for 96 h. Cells were then replated for MTT assays at the indicated periods. B, CML cells were infected with lentiviral shRNF6 for 96 h. Cells were then replated for MTT assays at the indicated periods. C,

K562 and KBM5 cells were infected with shRNF6, pLVX-VIM, or both. Cell viability was then measured at indicated periods by MTT assay. D, K562 and KBM5 cells were infected with shRNF6, pLVX-VIM, or both for 96 h, followed by migration assay using Transwell.

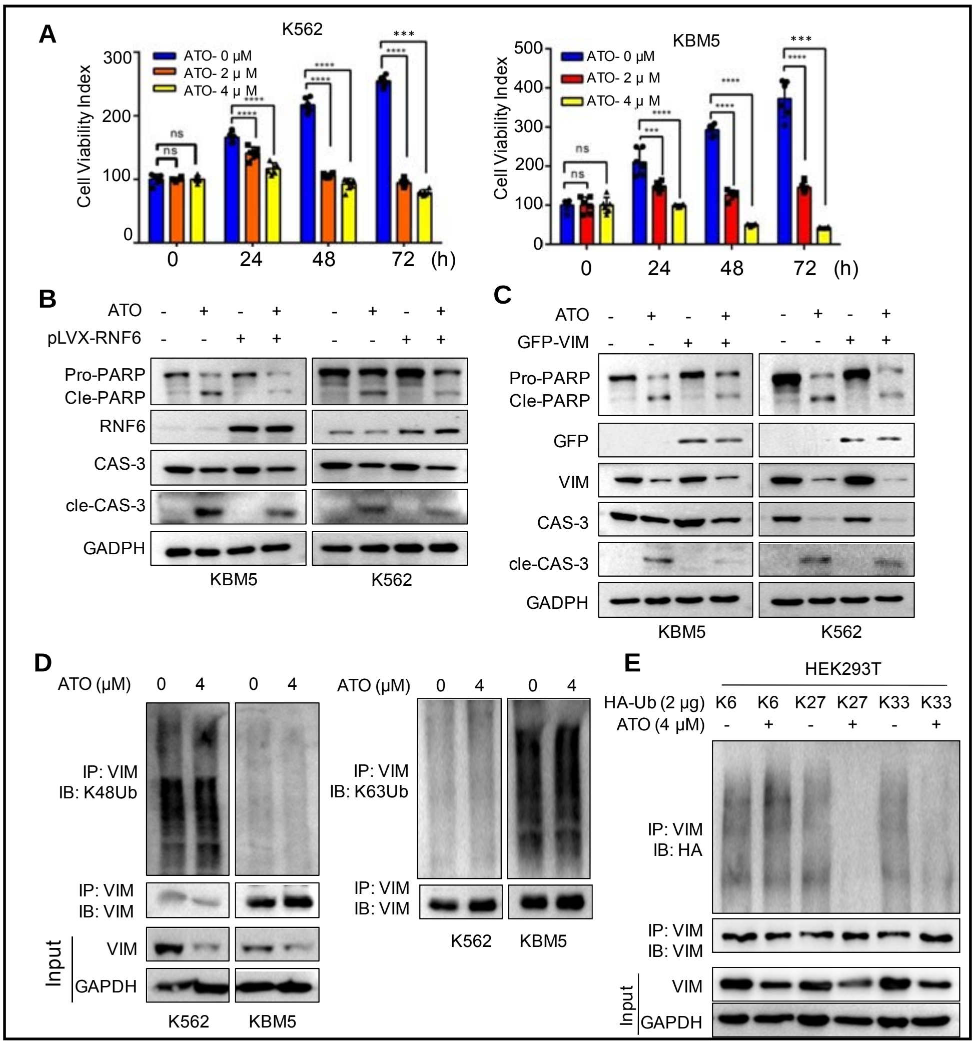

Supplemental Fig. 6. ATO induces CML cell apoptosis by downregulating RNF6 and VIM. A, CML cells were treated with arsenic trioxide (ATO) for indicated periods, followed by MTT assays. B, CML cells were infected with lentiviral RNF6 for 96 h, cells were then treated with ATO for 24 h. The cell lysates were then subjected to IB assays against PARP, Caspase-3 (CAS-3), and RNF6. C, CML cells were infected with lentiviral GFP-VIM for 96 h, cells were then treated

with ATO for 24 h. The cell lysates were subjected to IB assays against PARP, Caspase-3 (CAS-3), and VIM. D, CML cells were treated with ATO at indicated concentrations for 24 h, cell lysates were then subjected to immunoprecipitation/IB assays a K48-linked ubiquitination and K63-linked ubiquitination. E, HEK293T cells were transfected with HA-Ub-K6, -K27 or -K33 plasmids for 24 h, cells were then treated with ATO for 24 h. Cell lysates were then prepared for IP/IB assays as indicated.
